# Clinical pharmacology and regulatory consequences of GnRH analogues in prostate cancer

**DOI:** 10.1007/s00228-014-1682-1

**Published:** 2014-04-23

**Authors:** Niels Eckstein, Bodo Haas

**Affiliations:** Federal Institute of Drugs and Medical Devices, Kurt-Georg-Kiesinger-Allee 3, 53175 Bonn, Germany

**Keywords:** Androgen deprivation therapy (ADT), Flare-up, GnRH analogues, LHRH agonists, Therapeutic equivalence, Prostate cancer

## Abstract

**Introduction:**

GnRH (gonadotropin-releasing hormone) analogues are long-term known to be safe and effective in the clinical management of hormone-dependent advanced prostate cancer. However, their unusual mechanism of action of de-sensitizing pituitary receptors makes generic market entry challenging. In addition, safety aspects like initial flare-up, breakthrough escape, and miniflares render planning and organization of clinical registration trials a complex project.

**Regulatory requirements: therapeutic equivalence:**

Regulatory requirements are high as these medicines are compared to bilateral surgical castration with a 100 % success rate. GnRH analogues will be used probably even wider in the near future due to demographic development and extension of indications. However, they are challenged by their antagonistic counterparts, which are avoiding flare-up phenomena. The following article deals with regulatory requirements of GnRH analogues in regard to their clinical characteristics.

## Introduction

Prostate cancer is one of the most common malignancies worldwide and is a leading cause of morbidity and mortality in elderly men. In many countries of the industrialized world, it represents the most frequently newly diagnosed cancer in men [1]. According to data published by the American Cancer Society, more than 200,000 Americans were diagnosed with prostate cancer in 2013. Prostate cancer is the second leading cause of cancer deaths in the USA. It is estimated that about one in six men in the USA will be diagnosed with prostate cancer during their lifetime and 1 in 36 will die from this disease [2]. In Europe, there are more than 380,000 new cases each year, representing more than 22 % of cancers diagnosed in men. Prostate cancer is the most numerous cancer diagnosed in men and is the third most common cause of death through malignancy in men in Europe [3]. The precise etiology of prostate cancer is unknown, but in the overwhelming majority, its growth is dependent on testosterone as the main driver. However, testosterone itself is not responsible for oncogenic transformation but the main driver after the disease has occurred. Consequently, androgen deprivation causes remission or clinical improvement in a high proportion of locally advanced or metastatic prostate cancer. Approximately 75 % of patients with metastatic prostate cancer present tumor response to initial endocrine therapy. Antiandrogenic endocrine therapy is therefore the first and primary tool of clinicians in systemic treatment of patients with metastatic prostate cancer. Thus, GnRH (gonadotropin-releasing hormone) analogues play a pivotal role in clinical management of higher stages (T3–T4) of prostate cancer.

## Mechanism of action and indications of GnRH analogues

**Fig. 1:**
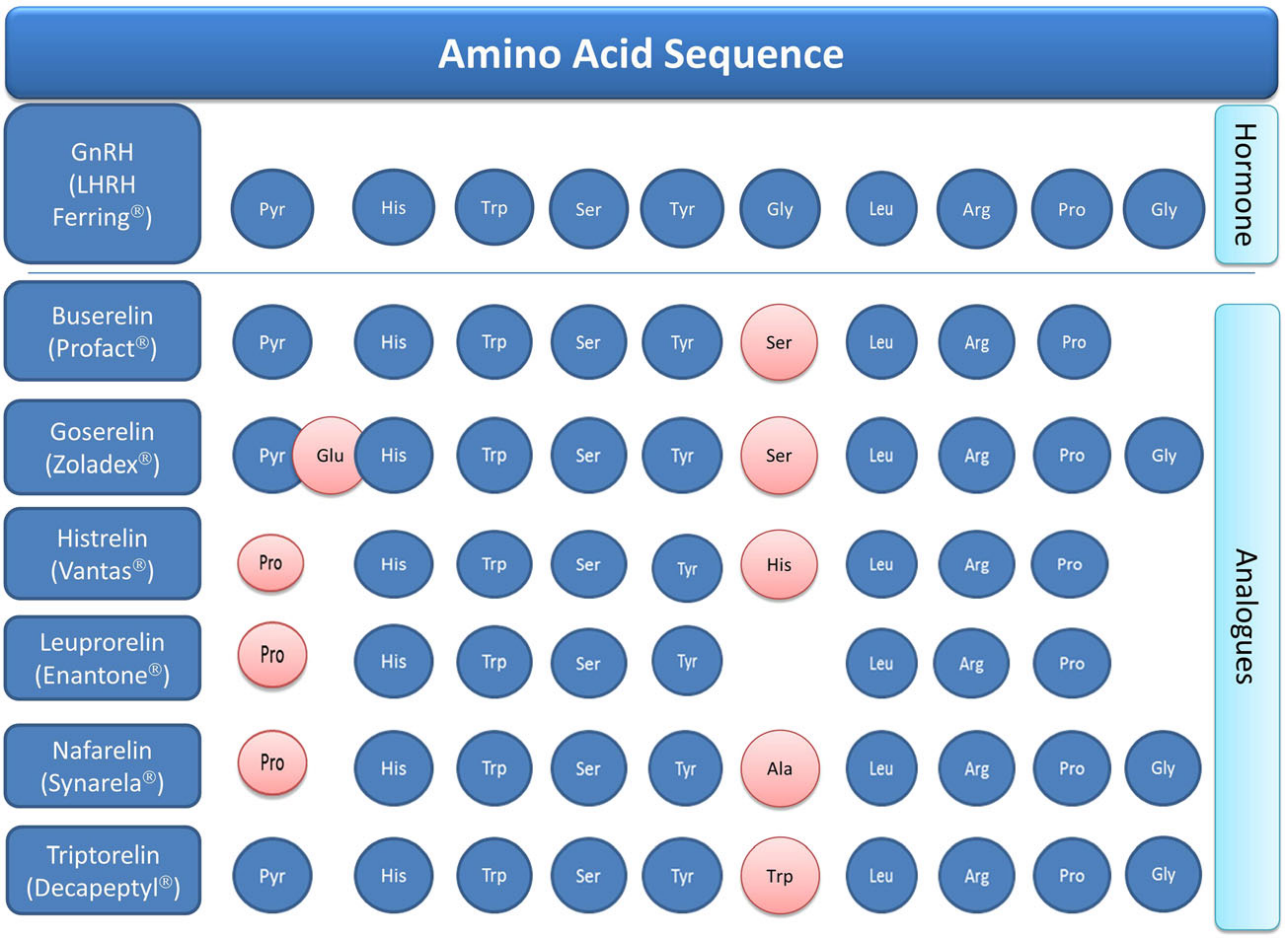
Alignment of the primary protein structure of GnRH analogues compared to the endogenous hormone

GnRH agonists are synthetic analogues of gonadotropin-releasing hormone (synonymous: GnRH, gonadorelin, luteinizing hormone-releasing hormone (LHRH)). The different substances are chemically synthesized usually in the form of a Merrifield synthesis [4] and are, therefore, not biological medicinal products. Thus, from a regulatory perspective, the overarching guideline for biosimilars does not apply for generic market entry [5]. An alignment of their amino acid sequence compared to the endogenous hormone and general information on licensed substances are provided in Fig. 1 and Table 1. Endogenous GnRH is released in a pulsatile manner approximately every 1–2 h. When given as a bolus in a nonretarded formulation, GnRH analogues like the hormone itself induce gonadotropin (luteinizing hormone (LH) and follicle-stimulating hormone (FSH)) secretion and, subsequently, stimulation of gonadal function. In contrast, long-term treatment with depot formulations causes downregulation and de-sensitization of GnRH receptors in the pituitary gland. As a consequence, the levels of testosterone in men and estradiol in women, respectively, are diminished. In men, LH released from the anterior pituitary gland stimulates the testes to produce testosterone. In women, FSH and LH cause the production of estrogen and progesterone to control the female cycle.

**Table 1:** General information on GnRH analogues

INN name (originator’s name)	ATC code	Galenic formulation (1 month)	Market entry	Amino acid sequence (and chemical molecule variations)	
GnRH (LHRH Ferring®)	H01 CA 01	Solution for injection	1983	Pyr-His-Trp-Ser-Tyr-Gly-Leu-Arg-Pro-Gly	Hormone
Buserelin (Profact®)	L02 AE 01	Implant (depot)	1988	Pyr-His-Trp-Ser-Tyr-Ser-Leu-Arg-Pro	Analogues
Goserelin (Zoladex®)	L02 AE 03	Implant (depot)	1990	Pyr-Glu-His-Trp-Ser-Tyr-D-Ser(But)-Leu-Arg-Pro-Azgly	
Histrelin (Vantas®)	L02 AE 05	Implant (depot)	2008	5-Oxo-Pro-His-Trp-Ser-Tyr-Ntbenzyl-d-His-Leu-Arg-*N*-ethyl-l-prolinamid	
Leuprorelin (Enantone®)	L02 AE 02	Microcapsules (retard)	1987	5-Oxo-Pro-His-Trp-Ser-Tyr-d-Leu-Arg-*N*-ethyl-l-prolinamid	
Nafarenlin (Synarela®)	H01 CA 02^a^	Nasal spray	1995	5-Oxo-Pro-His-Trp-Ser-Tyr-3-(2-naphthyl)-d-Ala-Leu-Arg-Pro-Gly	
Triptorelin (Decapeptyl®)	L02 AE 04	Solution for injection (retard)	2001	Pyr-His-Trp-Ser-Tyr-d-Trp-Leu-Arg-Pro-Gly	

^a^Only benign female indications

Physiological LH release results in a transient surge in serum testosterone levels (flare-up). However, continuous, unphysiological stimulation of LH secretion over extended periods of time by either repeated administration of GnRH or single administration of long-acting GnRH analogues results in downregulation of pituitary GnRH receptors by which gonadotropin secretion is dramatically reduced, resulting in gonadal suppression, with decreased testosterone production in men and estradiol production in women.

Accordingly, due to the inhibition of LH secretion by chronic administration, GnRH analogues are effective in the treatment of androgen-dependent prostate cancer in men [6] and hormone-dependent breast cancer in pre- and perimenopausal women [7]. In addition, GnRH analogues have a useful role in the management of some benign estrogen-dependent gynecological disorders like endometriosis and uterine fibroids [8]. However, pharmacokinetics (PK) are quite different in women compared to men [9]. Thus, from a regulatory perspective, clinical data recorded in male patients cannot justify an approval of female indications, irrespective of whether it is a benign or malign indication.

## Pharmacokinetics of GnRH analogues

Given that most clinical evidence was gathered with goserelin, the following characteristics are described for the originator Zoladex®. However, in different countries, different preferences for certain GnRH analogues exist [10]. After administration of the 3-month (3 M) goserelin implant at a dose of 10.8 mg, a maximum concentration (*C*_max_) of 8 to 10 ng/mL is reached within 2 h (*t*_max_) after administration with a second peak plasma concentration 7 weeks after administration of the implant.

Plasma protein binding of goserelin is low, ranging between 20 and 30 %. The apparent volumes of distribution determined after subcutaneous administration of a rapid-release aqueous solution were 44 ± 13 L for males and 20 ± 4 L for females, respectively, suggesting a distribution into total body water. However, this points to substantial differences in PK parameters between men and women.

Hydrolysis of the C-terminal amino acids is the major clearance mechanism of goserelin and other GnRH analogues. The metabolism of goserelin in humans is comparable to the profile of metabolites from animal studies: All metabolites found in humans have also been detected in preclinical animal studies. Clearance of goserelin following subcutaneous administration of the rapid-release solution formulation is very rapid. It is excreted primarily in urine. More than 90 % of the dose is recovered in urine and only 2 % in feces over a 5-day collection period. More than 75 % of the dose is excreted within 12 h.

An elimination half-life (*t*_1/2_) of 4.2 h is observed in healthy volunteers. In patients with prostate cancer, a similar *t*_1/2_ value of 4.6 h is observed. The goserelin pharmacokinetic/pharmacodynamic (PK/PD) relationship (measured using the originator Zoladex®) is nonlinear (on-off response) and time-dependent due to the de-sensitization of the pituitary gland which is maintained with very low levels of goserelin once achieved. However, the exact concentration required to de-sensitize these receptors is not known. Thus, formal demonstration of bioequivalence alone is not meaningful to file marketing authorization applications (MAA) for generic GnRH formulations.

## Regulatory requirements: therapeutic equivalence

According to their extraordinary mechanism of action, exceptional rules apply when generic formulations of GnRH analogues enter the market. As these substances mechanistically exert their effect by receptor de-sensitization, formal demonstration of bioequivalence is not meaningful in this context. GnRH itself physiologically is released in a pulsatile manner with an incretion burst approximately every 2 h (60–120 min). In contrast, GnRH analogues, also referred to as “super agonists,” stimulate the receptor over extended periods of time. This unphysiologically long stimulation causes an endocrine counteraction: GnRH analogues in this regard are a homogenous class of medicines exerting their effects by de-sensitization of receptors in the anterior pituitary gland rather than by single substance effects. As a consequence, in male patients, plasma testosterone is diminished to castration levels. Thus, reaching and maintaining castration levels of testosterone is the accepted surrogate in generic registration trials. Given this, it is intuitive that MAA cannot be filed under Article 10(1) of Directive 2001/83/EC (generic application) which would be entailed with formal demonstration of bioequivalence. In this class of substances, Article 10(3) (hybrid application) is applied. Thus, instead of bioequivalence, therapeutic equivalence is required at least by European agencies. This is somehow different in parts of the so-called pharmerging markets (i.e., South and Southeast Asia and Brazil, where formal demonstration of bioequivalence of the active substance is required). With respect to testosterone-lowering treatment options, orchiectomy (bilateral surgical castration) is considered the state-of-the-art treatment for androgen deprivation therapies (ADT). Thus, pharmacological therapies are compared to success rates of surgical castration [11]. It is thereby easy to understand why pivotal requirements for demonstrating therapeutic equivalence are so high: Usually, 90 % of patients must be successful in reaching and maintaining castration level (primary efficacy variable).

In this regard, it must be mentioned that “castration level” does not mean “zero level” could be reached. Ninety to ninety-five percent of endogenous testosterone is produced by the testes; however, a remaining source of the hormone derives from adrenal secretion (~5 %). Within the recent years, experts discussed whether lowering of the internationally accepted castration level would make sense. In detail, lowering from 0.5 to 0.2 ng/mL is under debate. From the medicinal perspective, this issue is discussed in view of safety and efficacy.

Various publications propagate lowering castration level for safety reasons [12]. Apart from theoretical discussions, prospectively collected clinical data suggest lowering this threshold [13]. This ongoing discussion also is mentioned in medicinal guidelines. The *Guidelines on Prostate Cancer* of the European Association of Urology (EAU, 2014) in this regard states (p. 96) [11]:

12.2.1 Castration level

Surgical castration is still considered the ‘gold standard’ for ADT, against which all other treatments are rated. It leads to a considerable decline in testosterone levels and induces a hypogonadal status, known as the ‘castration level’. The standard castrate level is < 50 ng/dL. It was defined more than 40 years ago, when testosterone level testing was limited. Current testing methods using chemiluminescence have found that the mean value of testosterone after surgical castration is 15 ng/dL. This has led to a revisiting of the current definition of castration, with a more appropriate level defined as below 20 ng/dL (1 nmol/L).

From the regulatory perspective, lowering testosterone castration level would have tremendous impact on generic marketing applications. Case numbers would have to rise substantially to manage the new challenge. However, it remains questionable whether the innovator products themselves would be successful: In the late 1980s, when those substances entered the market, 0.2 ng/mL was not in the discussion, and analytical methods to quantify testosterone blood levels post hoc nowadays are highly questionable.

## Other challenges in the approval process

### End-of-dose phenomenon (acute-on-chronic phenomenon)

**Fig. 2:**
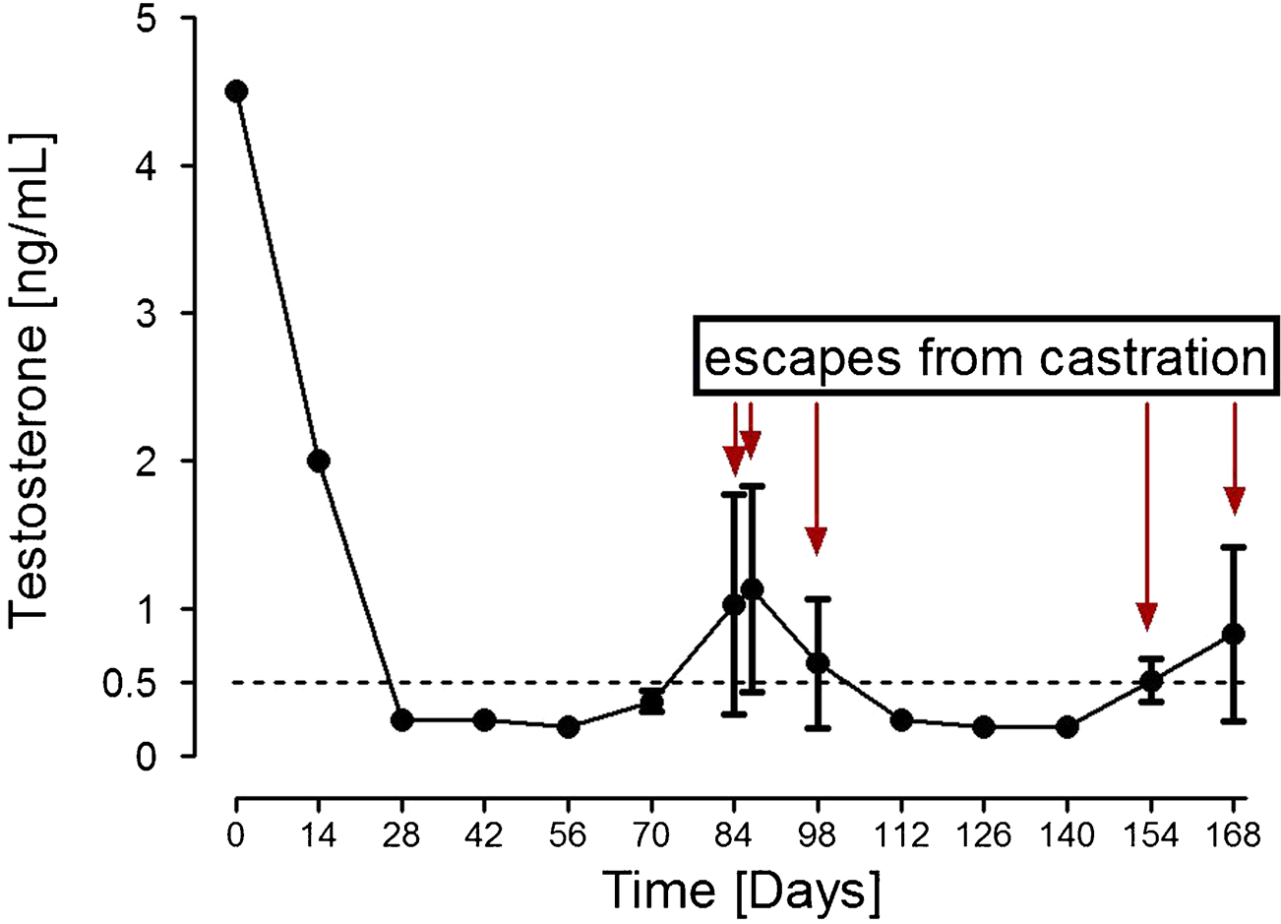
Premature exhaustion of a 3-month (3 M) depot formulation of a given GnRH analogue as example of the end-of-dose phenomenon, also called acute-on-chronic phenomenon (simulated curve)

**Fig. 3:**
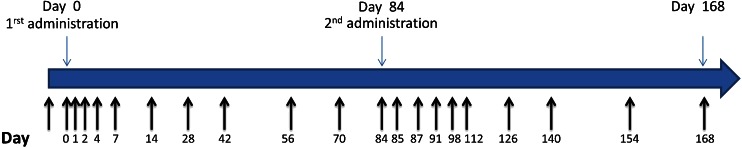
Possible schedule of blood sampling times to assess an end-of-dose phenomenon for a 3-month (3 M) depot formulation of a given GnRH analogue

End-of-dose phenomena in the context of GnRH analogues describe premature exhaustion of a depot formulation. As a consequence, testosterone levels rise at the end of the dosage interval (Fig. 2). In regard to the end-of-dose phenomenon, regulators usually will advise dense dose sampling especially at the end of each dosing interval. To this end, a single-dose study will not be sufficient in a registration trial, and at least two administrations of the study drug will be required because otherwise an acute-on-chronic phenomenon cannot be excluded. Thus, a possible schedule could look like the one indicated in Fig. 3.

**Fig. 4:**
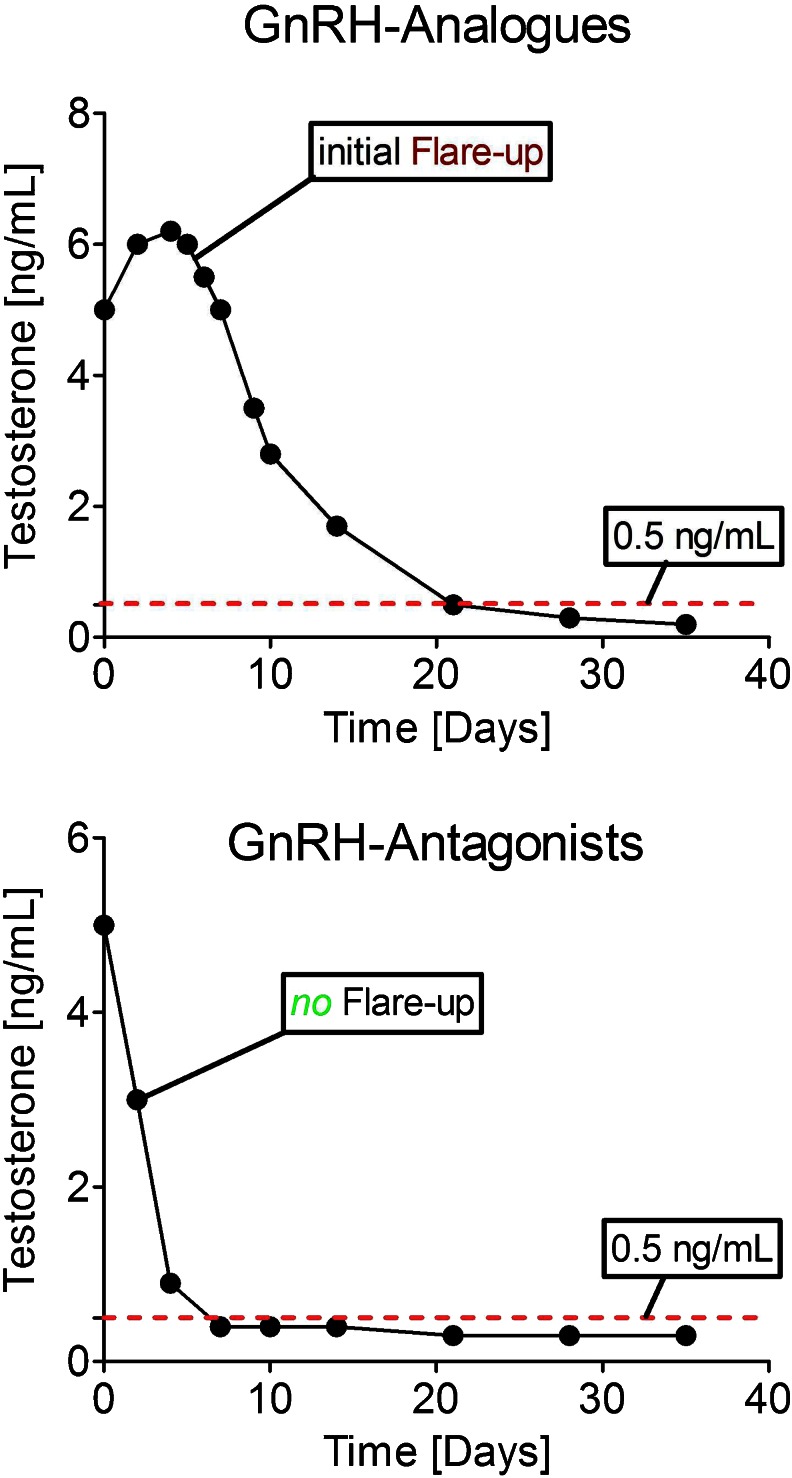
Comparison of testosterone time course under therapy with GnRH analogues (*upper graph*) and GnRH antagonists (*lower graph*), respectively. In regard to initial flare-ups, concomitant therapy with androgen receptor antagonists (flutamide, bicalutamide) is recommended within the first 3 weeks of GnRH analogue therapy

## Breakthrough escapes and miniflares

Along with the Vantas® referral [14], at first sight, things seemed to become easier for generic market applications. The European Medicines Agency (EMA) denied that a comparator arm would be pivotal in a registration trial pointing at the a.m. biochemical surrogate and an internationally accepted (and harmonized) threshold for castration. However, an in-depth analysis of the situation uncovers dangerous traps and pitfalls for applicants and CROs. Waiving the comparator arm is at the applicant’s risk. In this regard, a closer look at *breakthrough escapes* and *miniflares* is necessary.

Both are abruptly occurring transient escapes from castration under ADT. The discussion on these phenomena is two-edged as on the one hand, they seem to be rare. On the other hand, throughout the last decade, the particular hazardousness of rising testosterone under therapy was uncovered. Back in 2004, Chen et al. already mentioned that “… These data… imply that (even) *a modest increase in receptor concentration permits the receptor to function despite the lower levels of androgens in castrated patients*…” [15]. Attard et al. in 2009 discussed that pharmacological castration might hypersensitize prostate cancer cells to remaining steroid concentrations in patients’ blood [16]. This finding especially emphasizes that breakthrough escapes and/or miniflares might be of eminent hazardousness for patients. In this context, it is under debate whether the castration threshold must be lowered to switch off mitogenic signaling of the androgen receptor (AR). Various molecular mechanisms of hypersensitization are known today, such as overexpression of AR-mRNA or mutations of the receptor. In 2012, Galsky et al. summarized these findings: “Prostate cancer that progresses despite castrate levels of serum Testosterone have been historically referred to as ‘hormone-refractory’ or ‘androgen-independent’ disease. Among the most significant advances in prostate cancer over the past decade has been the realization, that these tumors are not in fact hormone refractory, *but may instead be hormone ultrasensitive*” [17]. In this context, the US-American guidelines of the National Comprehensive Cancer Network [18] point to the following recommendations (p. 21): “Androgen receptor activation and autocrine/paracrine androgen synthesis are potential mechanisms of recurrence of prostate cancer during ADT (ADT, Androgen Deprivation Therapy). Thus, castrate levels of testosterone should be maintained while additional therapies are applied.” Taken together, it is currently agreed by the scientific community that breakthrough escapes and miniflares are particularly dangerous and should be avoided in daily patient care.

However, they are clearly differentiated from insufficient galenic quality of the respective drug product which might end up in premature exhaustion of the administered depot (irrespective of whether it is an implant or consists of microcapsules). However, from the regulatory point of view, both are regarded as therapy failures with respect to the primary endpoint and might endanger the outcome of the whole trial as 90 % success rate easily is fallen below. Thus, one possible way to circumvent this pitfall would be to enroll at least a small comparator arm with complete pharmacokinetic characterization. Breakthrough escapes and miniflares as well as an ongoing discussion on lowering the castration level to 0.2 ng/mL were not an issue at the time the originator product entered the market. Thus, their performance in these contexts simply is not completely known. Taken together, waiving the comparator arm according to the Vantas® referral undoubtedly is a legal option but associated with the described risks.

Box: abarelix and degarelix antagonists without flares?

**Table:** 

A comparatively new treatment concept are GnRH antagonists. For instance, degarelix (Firmagon®) was licensed via the central European approval procedure [19]. Abarelix (Plenaxis®) was approved by the FDA in 2003. Compared to GnRH analogues, the castration level usually is reached without initial testosterone flare-up within 1 week (+antiandrogen flare protection) in the relief of lower urinary tract symptoms secondary to prostate cancer: results from a phase IIIb study (NCT00831233) [20]. In clinical trials, one third of patients even reached castration level after 3 days. Since pituitary receptors are neither downregulated nor de-sensitized, normalization of the testosterone level after termination of therapy is reached more rapidly and side effects resolve faster. However, safety profiles of antagonists are not completely known at present. Antagonists show more injection side reactions, and anaphylactoid reactions with urticaria, hypotension, pruritus, and syncopes were observed in 1.1 %. To this end, more clinical post-authorization data are needed to come to a final conclusion.
An initial flare-up effect (Fig. 4) may cause serious symptoms such as worsening of bone pain, ureteral obstruction, and spinal cord compression [21, 22]. Thus, antagonists may be of clinical benefit especially in metastasized patients.

## A regulatory outlook: current status and regulatory prospects

Localized prostate cancer describes a stage where the tumor is refined to the prostate and did not grow through the organ’s capsule. It is staged cT1–T2 (and N0 M0) according to EAU guidelines. Depending on TNM staging, watchful waiting (WW), active surveillance (AS), or radical prostatectomy (RP) might be an option and ADT is not indicated (or approved). However, throughout the recent years, earlier clinical use of ADT has emerged in many countries all over the world. Currently, it is questionable whether ADT in earlier stages of prostate cancer is of clinical benefit. A growing body of evidence seems to be in favor of this new concept, but mature long-term data on clinical outcomes are not yet available as summarized by Akaza [23]. According to their licensing status, GnRH analogues become an option when the disease already is in advanced stage (T3 or higher). Depot formulations of GnRH analogues have been used to treat hormone-dependent advanced prostate cancer for more than two decades now. It is undoubted that these substances are effective and a worthy tool for clinicians to (reversibly) castrate patients. Current data from the public domain also suggest a combination of ADT and external-beam radiotherapy (RT). However, some GnRH analogues are approved for combination treatment (triptorelin, leuprorelin) via national and de-central European procedures, others not (yet). As combination treatment already is recommended by European and US-American medicinal guidelines of high evidence, currently, there is a gap between clinical use and regulatory status of these substances. Management of locally advanced prostate cancer deals with both local control and the need to treat micrometastases undetectable with imaging techniques. Therefore, a multimodal strategy should apply as reflected in medicinal guidelines in Europe and the USA. The major treatment modalities currently proposed by the EAU in the treatment of locally advanced prostate cancer are

- Radical prostatectomy (RP)
- Definitive radiotherapy (DR)
- Hormonal ablation therapy

or a combination of these. Various clinical studies have addressed whether a combined treatment of ADT and RT is of clinical benefit.

Widmark et al. conducted a randomized, open phase III study comparing endocrine therapy with and without local radiotherapy, followed by castration on progression, in order to assess the effect of radiotherapy [24]. This trial included men from 47 centers throughout Scandinavia. Between 1996 and 2002, 875 patients with locally advanced prostate cancer (78 % staged T3 N0 M0) were enrolled to receive either endocrine treatment alone (3 months of total androgen blockade with leuprorelin followed by continuous endocrine treatment using flutamide (*n* = 439 patients)) or the same endocrine treatment combined with radiotherapy (*n* = 436 patients). The authors concluded that in patients with locally advanced prostate cancer, addition of RT to endocrine treatment halved the 10-year prostate cancer-specific mortality and substantially decreased overall mortality with acceptable risk of side effects compared with endocrine treatment alone.

Mottet et al. compared 3-year leuprorelin treatment (microsphere formulation) plus radiotherapy with leuprorelin alone in locally advanced prostate cancer patients [25]. This study was a multicenter, randomized, open controlled phase III trial in 264 histologically confirmed T3–T4 or pT3 N0 M0 prostate cancer patients randomized from March 2000 to December 2003. Patients received either 11.25 mg subcutaneous depot injection of leuprorelin every 3 months for 3 years plus external-beam radiotherapy (*n* = 133) or leuprorelin alone (*n* = 133). Flutamide for flare-up prevention (750 mg/day) was administered for 1 month. The authors concluded that combined therapy of leuprorelin and radiotherapy strongly favored improved PFS, locoregional control, and metastasis-free survival.

EORTC 22863 is a randomized phase III trial assessing the benefit of the addition of long-term androgen suppression with a GnRH analogues (the authors used goserelin for this study) to external irradiation in patients with prostate cancer with high metastatic risk (10-year follow-up) [26]. The authors concluded that in patients with prostate cancer with high metastatic risk, immediate androgen suppression with an GnRH analogue given during and for 3 years after external irradiation improves 10-year disease-free and overall survival without increasing late cardiovascular toxicity.

Apart from the a.m. studies, evidence in favor of combined therapy also is provided by the Radiation Therapy Oncology Group (RTOG) 85-31 [27], RTOG 86-10 [28], and RTOG 94-08 [29]. Apart from clinical studies, epidemiological trials also investigated this issue. Supporting studies on the effect of ADT in combination with RT are the following:

- D’Amico et al. [30]
- Hanks et al. [31] and Horwitz et al. [32]
- Bolla et al. [33]
- Bekelman et al. [34]
- Crook et al. [35]
- Souhami et al. [36]
- Lawton et al. [37]

In addition, clinical studies are underway to investigate whether long-term, short-term, or intermittent ADT is the best option with regard to safety issues: GnRH analogues are known to cause bone loss (eventually entailed with fractures). RTOG 92-02 especially, published by Hanks et al. in 2003, investigated this issue [31]. However, final judgment is not yet possible, and this discussion is ongoing.

## Conclusions

The current regulatory status of GnRH analogues is best described as “in between state.” The traditional approach of GnRH analogues as palliative medicines in locally advanced or metastatic situations is currently challenged by clinical practice to initiate ADT in earlier stages of the disease. At the other end of the scale, ADT in advanced prostate cancer is on its way to be widened (for not to say “flared”) also to comprise combined ADT and RT as a multimodal approach. Thus, GnRH analogues throughout the upcoming years will remain important in clinical management of prostate cancer. However, regulatory requirements are tremendous. For granting marketing authorizations, it is pivotal to demonstrate therapeutic equivalence for generic formulations on the basis of testosterone suppression (currently 0.5 ng/mL) to castrate levels in 90 % of cases or more. Galenic formulations must be able to avoid end-of-dose premature exhaustion, and receptor de-sensitization must be complete to avoid breakthrough escapes and/or miniflares. Whether GnRH antagonists will be a clinical alternative or even substitute GnRH analogues remains to be elucidated on the basis of clinical long-term follow-up data. Taken together, clinical evaluation and filing MAA will remain a huge challenge for the pharmaceutical industry in this highly complex and regulated field.

## Abbreviations

ADT: Androgen deprivation therapy
AR: Androgen receptor
AS: Active surveillance
*C*_max_: Maximal plasma concentration
DR: Definitive radiotherapy
EAU: European Association of Urology
EMA: European Medicines Agency
EORTC: European Organization for the Research and Treatment of Cancer
FSH: Follicle-stimulating hormone
GnRH: Gonadotropin-releasing hormone
LH: Luteinizing hormone
LHRH: Luteinizing hormone-releasing hormone
MAA: Marketing authorization application
PK: Pharmacokinetics
RP: Radical prostatectomy
RT: Radiotherapy
RTOG: Radiotherapy Oncology Group
*t*_1/2_: Elimination half-life
WW: Watchful waiting
